# Intestine-Specific Mttp Deletion Increases the Severity of Experimental Colitis and Leads to Greater Tumor Burden in a Model of Colitis Associated Cancer

**DOI:** 10.1371/journal.pone.0067819

**Published:** 2013-06-21

**Authors:** Yan Xie, Hitoshi Matsumoto, ILKe Nalbantoglu, Thomas A. Kerr, Jianyang Luo, Deborah C. Rubin, Susan Kennedy, Nicholas O. Davidson

**Affiliations:** 1 Department of Medicine, Washington University School of Medicine, St. Louis, Missouri, United States of America; 2 Department of Pathology and Immunology, Washington University School of Medicine, St. Louis, Missouri, United States of America; Virginia Tech, United States of America

## Abstract

**Background:**

Gut derived lipid factors have been implicated in systemic injury and inflammation but the precise pathways involved are unknown. In addition, dietary fat intake and obesity are independent risk factors for the development of colorectal cancer. Here we studied the severity of experimental colitis and the development of colitis associated cancer (CAC) in mice with an inducible block in chylomicron secretion and fat malabsorption, following intestine-specific deletion of microsomal triglyceride transfer protein (*Mttp-IKO*).

**Methodology/Principal Findings:**

*Mttp-IKO* mice exhibited more severe injury with ∼90% mortality following dextran sodium sulfate (DSS) induced colitis, compared to <20% in controls. Intestinal permeability was increased in *Mttp-IKO* mice compared to controls, both at baseline and after DSS administration, in association with increased circulating levels of TNFα. DSS treatment increased colonic mRNA expression of IL-1β and IL-17A as well as inflammasome expression in both genotypes, but the abundance of TNFα was selectively increased in DSS treated *Mttp-IKO* mice. There was a 2-fold increase in colonic tumor burden in *Mttp-IKO* mice following azoxymethane/DSS treatment, which was associated with increased colonic inflammation as well as alterations in cytokine expression. To examine the pathways by which alterations in fatty acid abundance might interact with cytokine signaling to regulate colonic epithelial growth, we used primary murine myofibroblasts to demonstrate that palmitate induced expression of amphiregulin and epiregulin and augmented the increase in both of these growth mediators when added to IL-1βor to TNFα.

**Conclusions:**

These studies demonstrate that *Mttp-IKO* mice, despite absorbing virtually no dietary fat, exhibit augmented fatty acid dependent signaling that in turn exacerbates colonic injury and increases tumor formation.

## Introduction

The continued epidemic of obesity and its associated comorbidities has fueled interest in understanding the pathways and mediators involved, particularly in relation to inflammation and cancer. Obesity and its related metabolic complications have been demonstrated to play a role in a number of cancers, including colorectal cancer (CRC) and consequently dietary modification and weight control are viewed as key modifiable risk factors [1]. However, the precise mechanisms and pathways by which obesity contributes to CRC risk are still incompletely understood and are likely to include alterations in systemic inflammation and cytokine signaling, insulin resistance as well as local and systemic effects of altered nutrient uptake including fatty acids, cholesterol and bile acids [2]. Because of its importance as an energy source, dietary fat intake has been a target for intervention as a strategy to mitigate the effects of obesity and inflammation on CRC risk [3].

In addition to the role for obesity and fat intake in modulating inflammation and CRC risk, there is emerging information suggesting that the small intestine plays an important role as a mediator in the inflammatory response to systemic sepsis [4], [5]. In this scenario, it has been suggested that the small intestine secretes lipid products derived from luminal digestion, which are then transported in mesenteric lymphatics, where they induce organ failure at distant sites [6], [7]. Other work has reinforced this gut-lymph hypothesis by demonstrating that ligation of the mesenteric duct (thereby eliminating delivery of chylomicrons from the intestine into the systemic circulation) abrogates the systemic effects of shock, findings that point to the importance of lipid mediators arising from the intestine in the pathogenesis of systemic inflammation [8], [9]. Other work has implicated lipoproteins as vehicles for transporting growth factors and morphogens, including mammalian Wnt3, suggesting a more fundamental role for lipid transport in the regulation of epithelial growth and proliferation [10].

In this study, we have examined the role of intestinal chylomicron secretion in order to understand the role of intestinal lipid transport in the pathogenesis of colonic inflammation and colitis associate cancer (CAC). We recently demonstrated that mice with conditional intestine-specific deletion of microsomal triglyceride transfer protein (*Mttp-IKO*) develop virtually complete block of intestinal absorption [11] and exhibit a survival advantage when challenged with *Pseudomonas aeruginosa*, the most common cause of gram negative pneumonia [12]. Those findings raised the possibility that blocking intestinal chylomicron secretion might also attenuate the effects of chemical induced experimental colitis and in turn abrogate colorectal cancer development following azoxymethane/dextran sodium sulfate (AOM/DSS) challenge [13]. Our findings, however, revealed that *Mttp-IKO* mice developed worse colonic injury and increased tumor burden. Furthermore, we found that altered fatty acid signaling may play a key role in promoting these phenotypes.

## Materials and Methods

### Animals

Mttp^flox/flox^ villin-Cre-ER^T2^ (*Mttp-IKO*) mice (along with littermate controls) were used for these studies, in a background of ∼75% C57BL/6 and ∼25% 129/SvJ. Cre recombinase expression in villus epithelial cells was induced by five daily intraperitoneal injections of 1 mg tamoxifen (Sigma), as described previously [11]. Experiments were performed on mice consuming a regular low fat rodent chow. Experimental colitis was induced by administration of 2.5% dextran sodium sulfate (DSS) (MW 40,000–50,000, Cat#9011-18-1, Affymetrix, Inc, Cleveland, Ohio.), for the indicated times in the figure legends and weighed daily. CAC was induced by injection of 8 week old mice with 10 mg/kg body weight azoxymethane (AOM, Sigma) followed by three cycles (5 days) of DSS starting at 5^th^(2%), 26^th^ (2.5%) and 46^th^ (2.5%) day after AOM injection, minor modifications of the protocol used by others [14]. Mice were sacrificed at 9 weeks after AOM injection and tissues collected for analysis. All animal studies were approved by the Animal Studies Committees of Washington University School of Medicine (#20100146) and were conducted in strict accordance with the National Institutes of Health guidelines for the use of laboratory animals.

### Histomorphological analyses

Samples of small intestine and colon were fixed and embedded for sectioning and hematoxylin and eosin (H&E) staining. Where indicated, intestinal sections were stained for lipid droplets using osmium tetroxide as described [12] Histologic scoring of DSS injury was undertaken and quantitated using the parameters described [15]. Intestinal proliferation was examined in mice injected with 5-Bromo-2′deoxyuridine (BrdU) (200 µl volume, 5 mg/ml diluted in normal saline; Sigma), 2 hours prior to sacrifice. Samples were processed for BrdU staining as described [12]. Analysis of colon tumor burden was undertaken using samples fixed in 10% formalin (Sigma) and pinned for scoring by an investigator blinded to genotype. Each sample was photographed using a Nikon SMZ800 dissecting microscope and a Photometrics CooLSNAPcf camera (Imaging Processing Services). The area of each section was determined and the size of tumors quantitated using Metavue software (Molecular Devices). H&E sections were also reviewed by a pathologist (IN), blinded to genotype.

### Intestinal permeability and cytokine assays

Intestinal permeability was determined following injection of FITC labeled dextran (FD-4, MW 4000, Sigma). Control and *Mttp-IKO* mice were gavaged with FD-4 (400 mg/Kg body weight), prior to and 7 days after 2.5% DSS treatment and sera were collected 4 hour later. Serum FITC was measured on a fluorimeter (Synergy HT, BioTek®) at excitation 485/20, emission 528/20. For LPS determinations, sera were diluted 1∶10 and heated at 70°C for 15 minutes to inactive inhibitors. LPS was then measured using the LAL chromogenic endotoxin quantitation kit according the manufacturer's instructions (ThermoScientific, Rockford, IL). Serum TNFα and IL-1β were quantitated by ELISA using kits purchased from BD Biosciences (San Jose, CA) or R&D systems (Minneapolis, MN) following the manufacturers' instructions.

### Real time quantitative polymerase chain reaction

Total RNA was isolated from intestinal tissues using Trizol either under standard protocols or for samples treated with DSS, using the modification described by Kerr and colleagues [16]. RNAs were quantitated using primer pairs (sequences available upon request) designed by Primer express software (Applied Biosystems). Relative mRNA abundance is expressed relative to control mice without DSS treatment, normalized to GAPDH.

### Colonic lipid content, Fecal lipid content and protein expression

Total lipids were extracted from feces as well as from proximal colon, as previously described [11]. Triglyceride (TG), total cholesterol (TC) and free fatty acid (FFA) were measured enzymatically using Wako kits (Wako Chemicals, Richmond, VA). Western blotting was conducted in standard fashion using antibodies whose source is indicated in the relevant figure legend. For colonic TNFα and IL1β content samples of descending colon were homogenized in buffer containing 20 mM Tris (pH 7.4), 1 mM Sodium orthovanadate, 150 mM NaCl, 1 mM EDTA, 1 mM EGTA, 1% Triton, 50 mM Sodium fluoride, 50 mM β-glycerophosphate and 1× Complete mini protease inhibitor cocktail (Roche, Mannhein, Germany). TNFα and IL1β levels were measured in clarified supernatants using the ELISA kits described above and normalized to protein concentration.

### Primary murine intestinal myofibroblasts

Myofibroblasts were isolated from C57BL/6 wild type mice as described [17] and 1×10^5^ cells per well were seeded in 6-well plates and cultured overnight. The phenotype of these cells was verified by positive staining for smooth muscle actin and vimentin and negative staining for desmin and cytokeratin [13], [17]. These myofibroblasts cells were then cultured for 12 hours in DMEM containing 0.25% FBA and 0.6% Fatty acid free bovine serum albumin (BSA, A8806, Sigma), supplemented with either buffer alone (BSA, Control), 200 µM Palmitate–BSA (PAL), TNFα at 100 ng/ml (TNFα) (R&D), IL1β at 10 ng/ml (IL1β) (R&D) or combinations of 200 µM Palmitate–BSA plus TNFα 100 ng/ml (TNFα+ PAL) or 200 µM Palmitate –BSA and IL1β 10 ng/ml (IL1β+ PAL), as indicated in the legend. These studies were all performed on myofibroblasts at passage 6–8. Sodium palmitate was conjugated with fatty acid free BSA at ratio of 2∶1 (Palmitate: BSA), to make a 3 mM palmitate-BSA stock solution. Total RNA was extracted from individual samples and mRNAs quantitated as described above.

### Statistical Analysis

Multiple group comparisons of continuous data sets were performed using one-way analysis of variance followed by post-hoc t-test using Prism 4.0 (GraphPad Software, San Diego, CA) and Microsoft Excel. Data are reported as means ± SEM. Survival studies were analyzed via the log-rank test. A p value <0.05 was considered to be statistically significant.

## Results

### Increased severity of DSS colitis in Mttp-IKO mice

**Figure 1:**
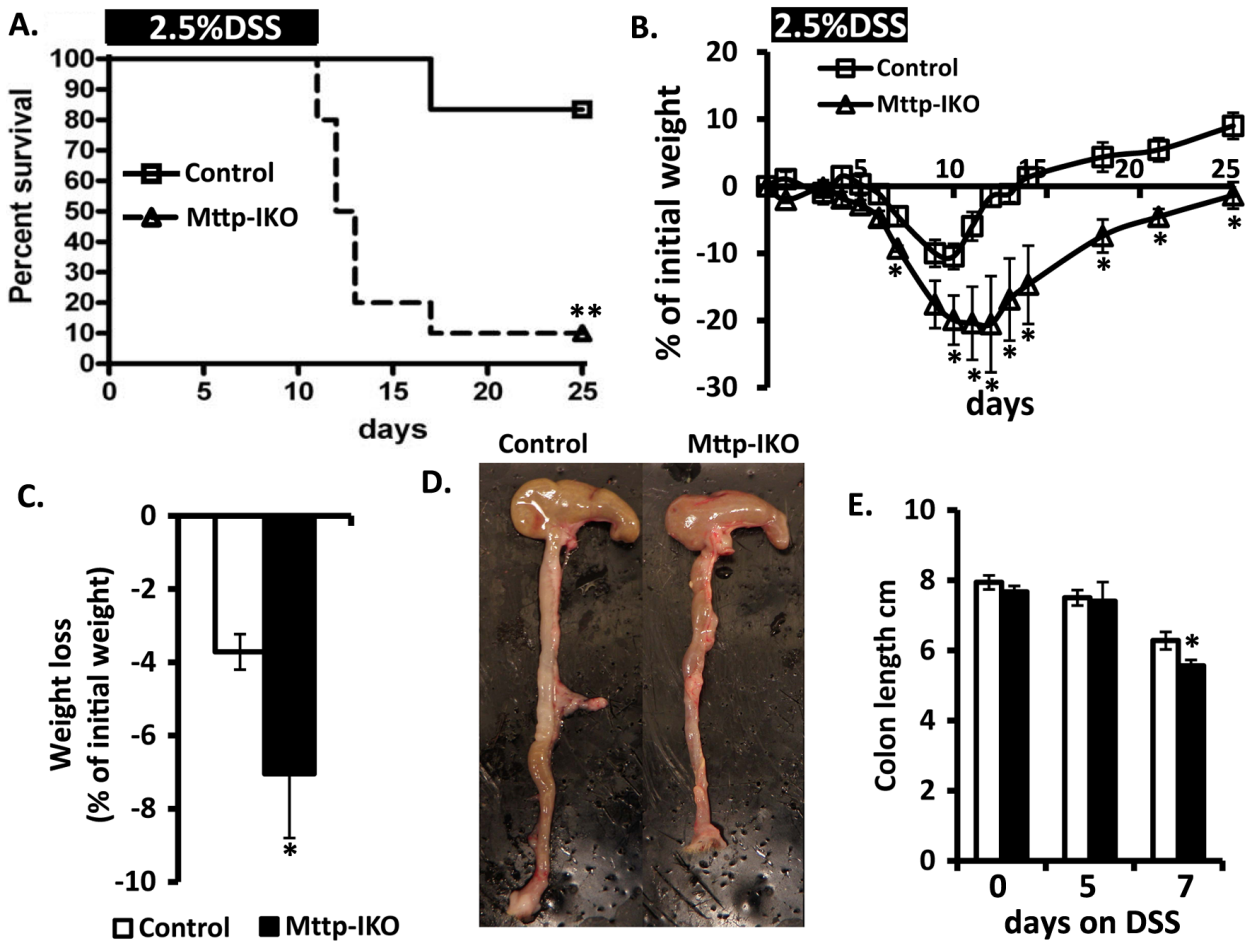
Increased colonic injury in DSS treated*Mttp-IKO* mice. **A**. Decreased survival of *Mttp-IKO* (n = 10) versus littermate controls (n = 12). 8–10 weeks mice were fed 2.5% DSS in drinking water for 12 days and followed up to 25 days. **p<0.01. **B**. Weight recovery curve after one cycle of DSS. Mice (n = 4 mice per group) were fed 2.5% DSS for 7 days and weighed every 1–3 days up to 25 days after the 1^st^ day of DSS treatment. *p<0.05. **C**. Weight loss after 7days DSS treatment, n = 10–12 mice per genotype. Data are presented as mean% ± SEM of initial weight. *p<0.05, **p<0.01 **D**. Representative images of gross appearance of colon from control and *Mttp IKO* mice after 7 days DSS treatment. **E**. Colon length at 0, 5 and 7 days on 2.5% DSS. Data are mean ± SE of 4–9 mice per group.*p<0.05.

**Figure 2:**
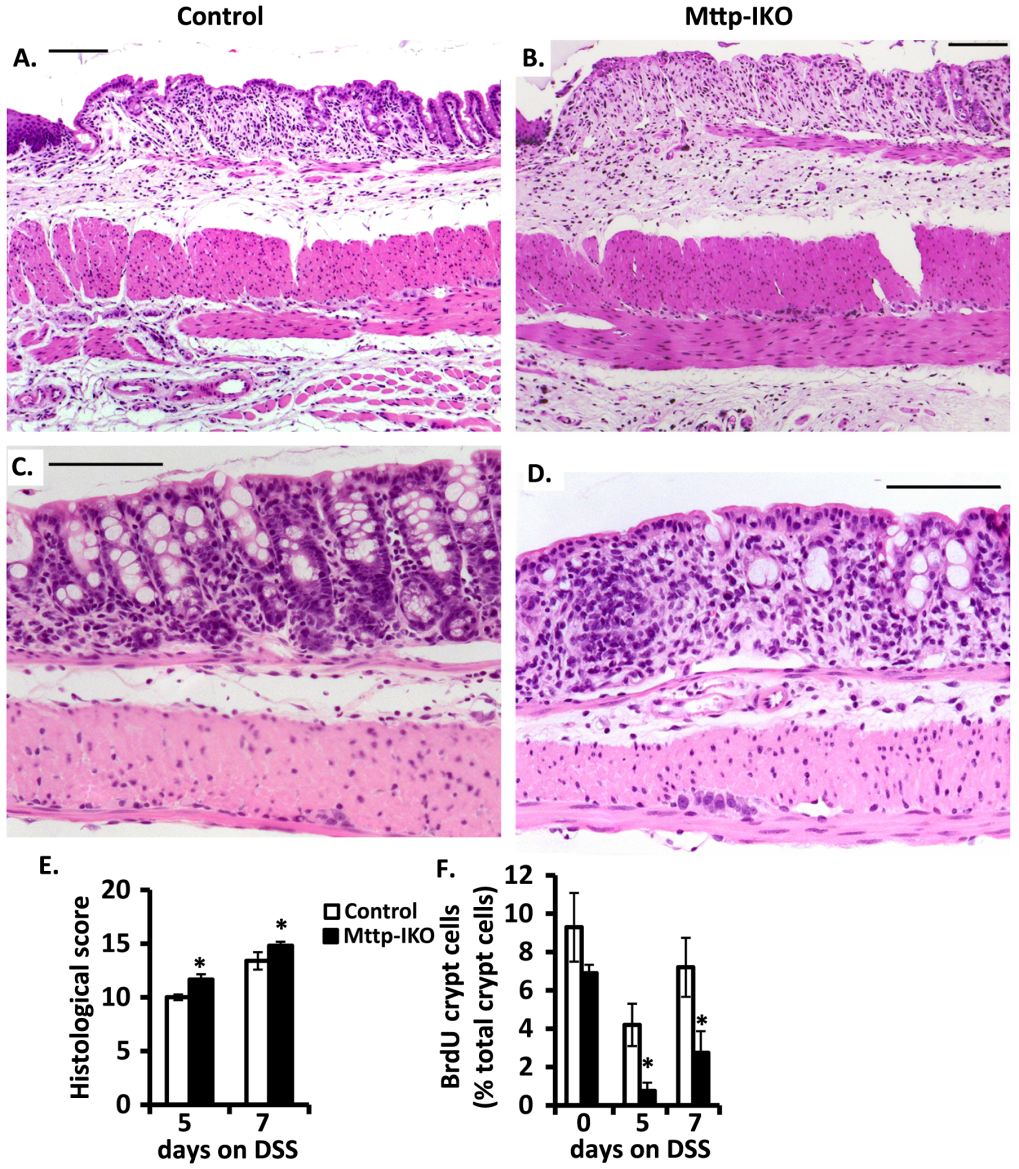
Increased inflammation and decreased proliferation in DSS-treated*Mttp-IKO* mice. **A**. and **B**. Representative histological images of comparable regions of distal colon from control (A.) and *Mttp-IKO* mice (B.) after 5 days of DSS treatment. Panels A and B show increased mucosal injury in *Mttp-IKO* mice characterized by increased lamina propria inflammation extending to the submucosa with loss of crypts. **C**. and **D**. Representative histologic images of comparable regions of descending colon from control (C.) and *Mttp-IKO* mice (D.) after 7 days DSS treatment. Panels C and D show an increase in lamina propria inflammation, focal cryptitis characterized by neutrophilic infiltration and focal crypt drop-out in *Mttp-IKO* mice. Bars indicate 100 µm. **E**. Quantitative estimate of histological damage (n = 5–7 mice per group). **F**. BrdU positive crypt cells in rectal mucosa. Data were the mean ± SEM of n = 5–6 mice per group. *p<0.05.

We administered 2.5% DSS in drinking water for 12 days in order to evaluate the impact on overall survival by genotype. Our findings revealed that *Mttp-IKO* mice exhibit accelerated death and markedly reduced survival (1/10) compared to control mice (9/11) Figure 1A. The augmented injury phenotype in *Mttp-IKO* mice was associated with greater weight loss in shorter (7 days) experiments (Figure 1C) and a delayed return to baseline weight (Figure 1B), along with reduced colon length (Figure 1D, E). There was also more severe histologic injury as evidenced by increased lamina propria inflammation and crypt drop-out in the descending colon in *Mttp-IKO* mice at both 5 days (Figure 2A, B, E) and at 7 days (Figure 2C, D, E) and reduced cellular proliferation as evidenced by decreased BrdU incorporation (Figure 2 F).

### DSS administration does not induce macroscopic small intestinal injury in Mttp-IKO mice and is not associated with colonic lipid accumulation

**Figure 3:**
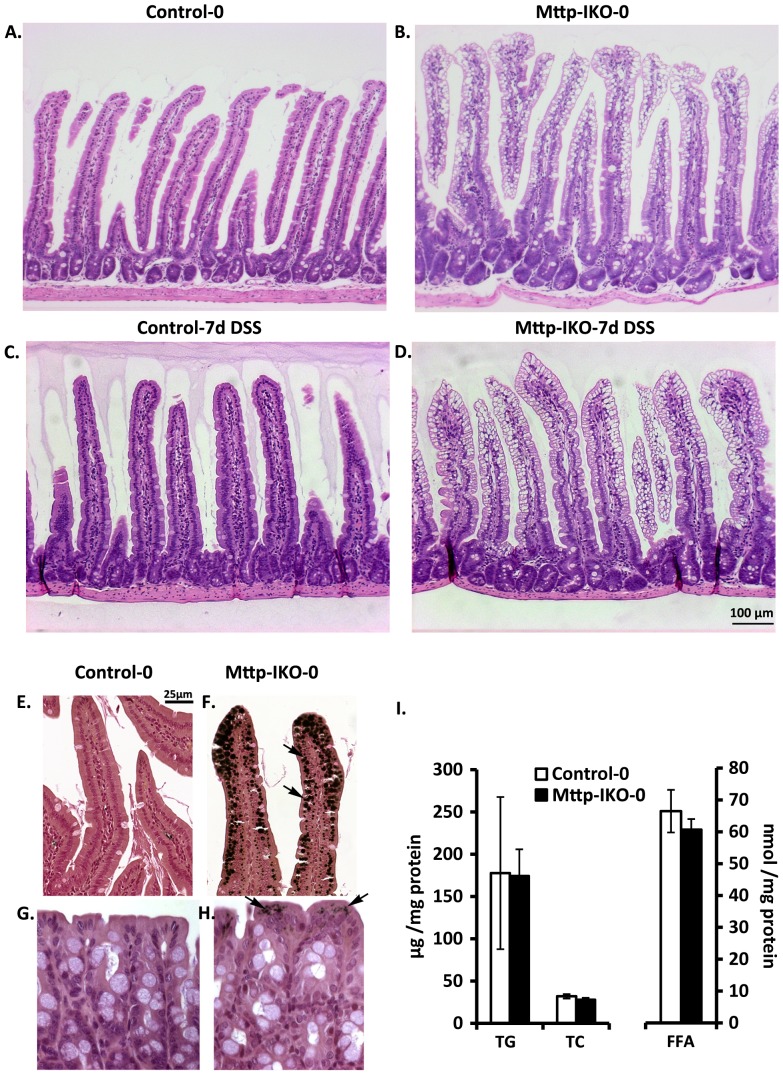
Morphology and lipid accumulation in intestine and colon of control and DSS treated*Mttp-IKO* mice. **A-D**. Representative H&E staining of small intestine from Control and *Mttp-IKO* mice either without DSS treatment (A. Control-0 and B. Mttp-IKO-0) or after 7 days DSS treatment (C. Control-7 and D. Mttp-IKO-7). Small intestine from *Mttp-IKO* mice (B and D) shows the villus lipid accumulation (vacuolar structure in H&E staining), but no significant injury or inflammation after 7 days DSS treatment (D vs B). **E-H**. Representative Osmium tetroxide staining of lipid droplets in small intestine (E and F) and Colon (G and H) without DSS treatment. Lipid droplets appear as brown images (arrows). Note the abundant lipids droplets in *Mttp-IKO* small intestinal enterocytes (F), by contrast with only scattered lipid droplets in *Mttp-IKO* colonic epithelial cells (H). **I**. Colonic lipids were extracted from control and *Mttp-IKO* mice without DSS treatment and lipid species (TG, TC and FFA) measured. The data are presented as the mean ± SEM of 4–5 mice per group.

Our previous studies demonstrated that blocking chylomicron secretion from the intestine of *Mttp-IKO* mice results in massive lipid engorgement with villus distortion [11], raising the possibility that the high mortality encountered following DSS administration might reflect gross damage in the small intestine of these mice. However, this was not the case. As seen in Figure 3 A-D, we observed the expected lipid accumulation in small intestinal villi in *Mttp-IKO* mice but there was no gross or histological evidence of ulceration in any region of the small intestine following 7 days of DSS treatment. In addition, since Mttp is expressed in the colon of mice (albeit at low levels [18], [19]) we explored the possibility that colonic lipid accumulation might contribute to the phenotype observed in *Mttp-IKO* mice. However, this again was not the case. As seen in Figure 3E-H, while we detected abundant lipid droplet accumulation in the small intestine of *Mttp-IKO* mice (as previously noted [11]) there were only rare, scattered osmium staining lipid droplets seen in the colon of *Mttp-IKO* mice and there was no accumulation of triglyceride, cholesterol or free fatty acid within colonic mucosa as detectable by enzymatic assay (Figure 3I).

### Increased intestinal permeability in Mttp-IKO mice and the role of an adaptive stress response

**Figure 4:**
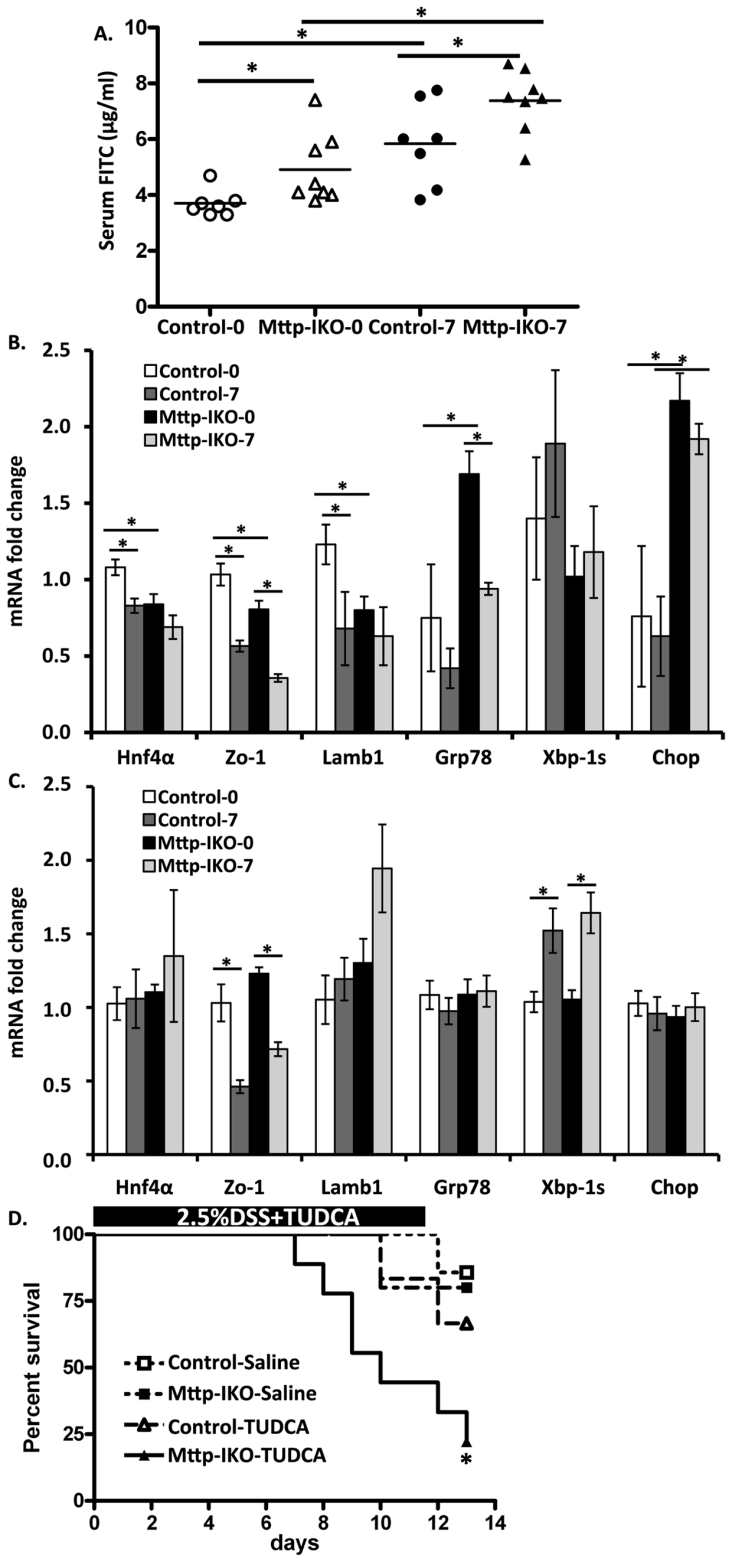
Impaired intestinal barrier and ER stress in*Mttp-IKO* mice. **A**. *Mttp-IKO* and control mice either before DSS or 7 days after DSS treatment were orally administered FITC-dextran and serum levels measured 4 hrs later. Data are the mean ± SEM of 7–8 mice per group.*P<0.05. **B. and C.** mRNA expression of genes related to ER stress and epithelial barrier functioin in small intestine and colon. mRNA expression was measured by QPCR and expressed as fold change compared to non-DSS treated control mice after normalized to internal control Gapdh. Data are presented as the mean ± SEM of 4–5 mice per group. **D**. The effect of TUDCA on survival proportion of Control and *Mttp-IKO* mice on DSS. 8–10 week old mice were treated with TUDCA or saline intraperitoneally during simultaneous administration of DSS (2.5%) in drinking water for 12 days. n = 5–9 each group. *p<0.05.

While the findings above imply that there are no gross alterations in villus integrity in *Mttp-IKO* mice, we explored the possibility that more subtle alterations might be associated with barrier dysfunction. To address this possibility, we administered FITC labeled dextran by gavage to mice of both genotypes, either before or after 7 days of DSS administration. Our findings revealed that serum FITC levels were significantly higher in *Mttp-IKO* mice under both conditions (Figure 4A). These findings suggest that there is altered barrier function at baseline in *Mttp-IKO* mice that becomes further impaired in the setting of DSS injury. We next considered the possibility that the increased intestinal permeability phenotype in *Mttp-IKO* mice might in part reflect alterations in the expression of integral membrane proteins involved in tight junction maintenance and whose altered expression has been linked to defects in human inflammatory bowel disease [20], [21]. We found that expression of HNF4α, ZO-1 and Lamb1 was decreased at baseline in the small intestine of *Mttp-IKO* mice and was decreased following DSS treatment, particularly in control mice (Figure 4B). There was a corresponding decrease in ZO-1 expression in the colon upon DSS treatment with similar responses in control and *Mttp-IKO* mice (Figure 4C).

We also examined parameters of the unfolded protein response (UPR) and integrated stress response pathways in the small intestine and colon of both genotypes and the impact of DSS exposure. Those findings demonstrated increased baseline expression of Grp78 and Chop in the small intestine of *Mttp-IKO* mice (Figure 4B), as previously noted [22]. However, DSS treatment failed to produce a further increase in any of these stress markers, but rather tended to reduce expression of Grp78 and Chop in the small intestine of *Mttp-IKO* mice (Figure 4B). By contrast, there were no baseline changes in UPR mRNAs in the colon of *Mttp-IKO* mice, but DSS treatment resulted in increased abundance of Xbp-1s in both genotypes (Figure 4C). These findings suggest that there is no baseline UPR response in the colon of *Mttp-IKO* mice. To examine the possibility that an adaptive ER stress response might influence the injury phenotype observed in *Mttp-IKO* mice, we treated mice with the chemical chaperone tauroursodeoxycholic acid (TUDCA) since previous studies have shown that this strategy attenuates the UPR in metabolic stress conditions in mice [22], [23]. These findings reveal that TUDCA treatment failed to rescue the severe phenotype of DSS treated *Mttp-IKO* mice and if anything led to accelerated death in these animals (Figure 4D). These findings together lead us to conclude that the features of ER stress and the UPR noted in the small intestine of *Mttp-IKO* mice are adaptive in nature and that abrogation of this response leads to worse injury.

### Enhanced colonic inflammation in Mttp-IKO mice in response to DSS injury

**Figure 5:**
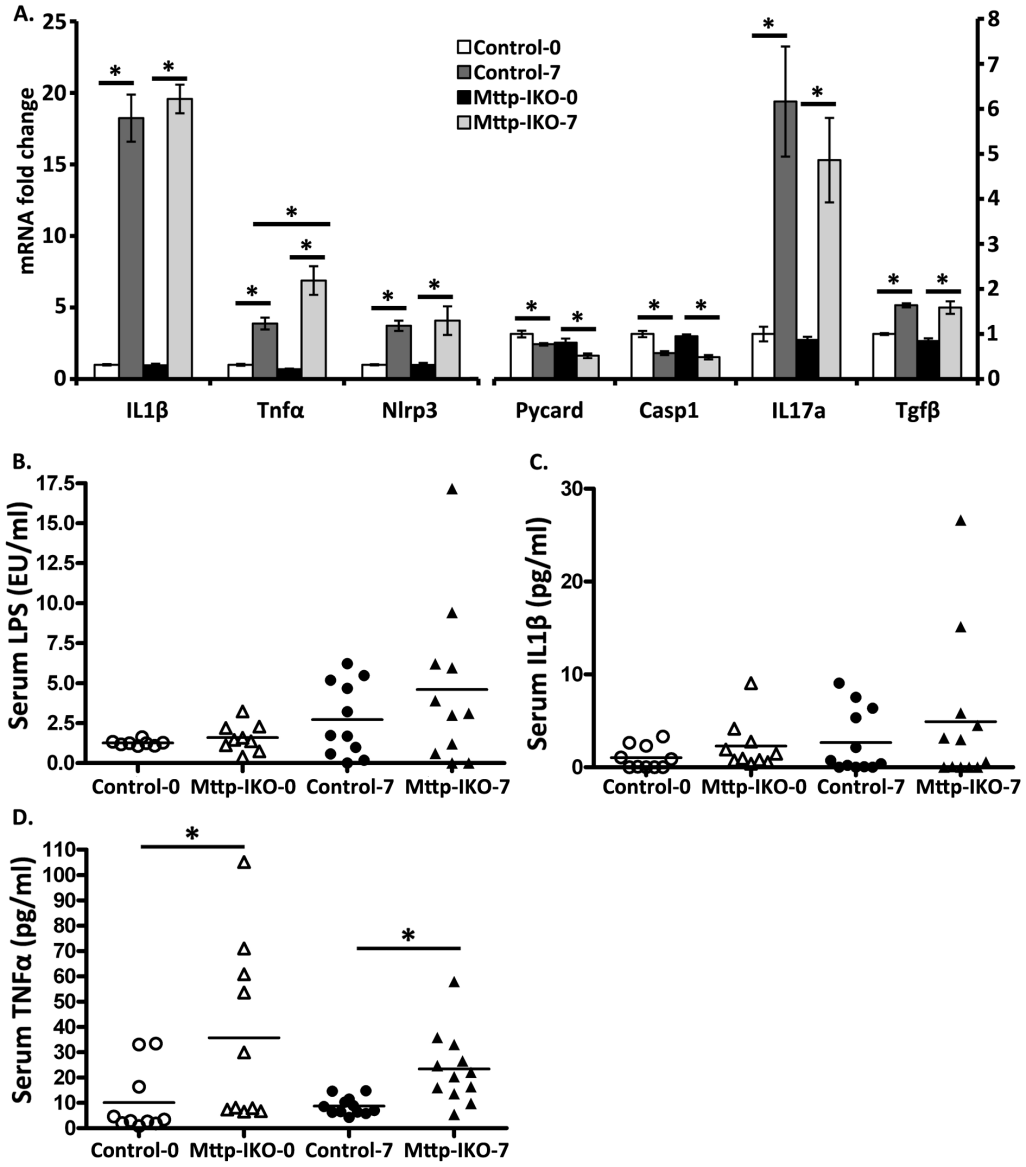
Increased colonic and systemic inflammation in*Mttp-IKO* mice. **A**. mRNA expression of genes related to inflammation in descending colon of control and *Mttp-IKO* mice before and after 7 days DSS treatment. mRNA was measured by QPCR and expressed as fold changes compared to non-DSS-treated control mice after normalized to Gapdh. Data are presented as the mean ± SEM of 5–9 mice per group. *p<0.05. **B.-D**. Serum cytokines in control and *Mttp-IKO* mice. Blood was collected from control and *Mttp-IKO* mice before or 7 days after DSS treatment. Serum LPS (**B**), IL1β (**C**) and TNFα (**D**) levels were measured as described in Methods. Data are the mean ± SEM of 8–10 mice per group. *p<0.05.

We observed the expected increase in expression of several cytokine mRNAs in the colon of DSS treated mice, including IL-1β, IL-17A and Nlrp3, with comparable induction in both genotypes (Figure 5A). However we observed a greater increase in TNFα expression in the colon of DSS treated *Mttp-IKO* mice compared to controls (Figure 5A). We also examined serum levels of LPS, IL-1β and TNFα, which have been previously associated with intestinal injury in mice treated with DSS [14], [24]. Those findings revealed that serum TNFα levels were consistently elevated in *Mttp-IKO* mice, both at baseline and following DSS treatment while serum levels of LPS and IL-1β were more variable (Figure 5B-D).

### Colonic injury and inflammation is associated with increased susceptibility to CAC in Mttp-IKO mice

**Figure 6:**
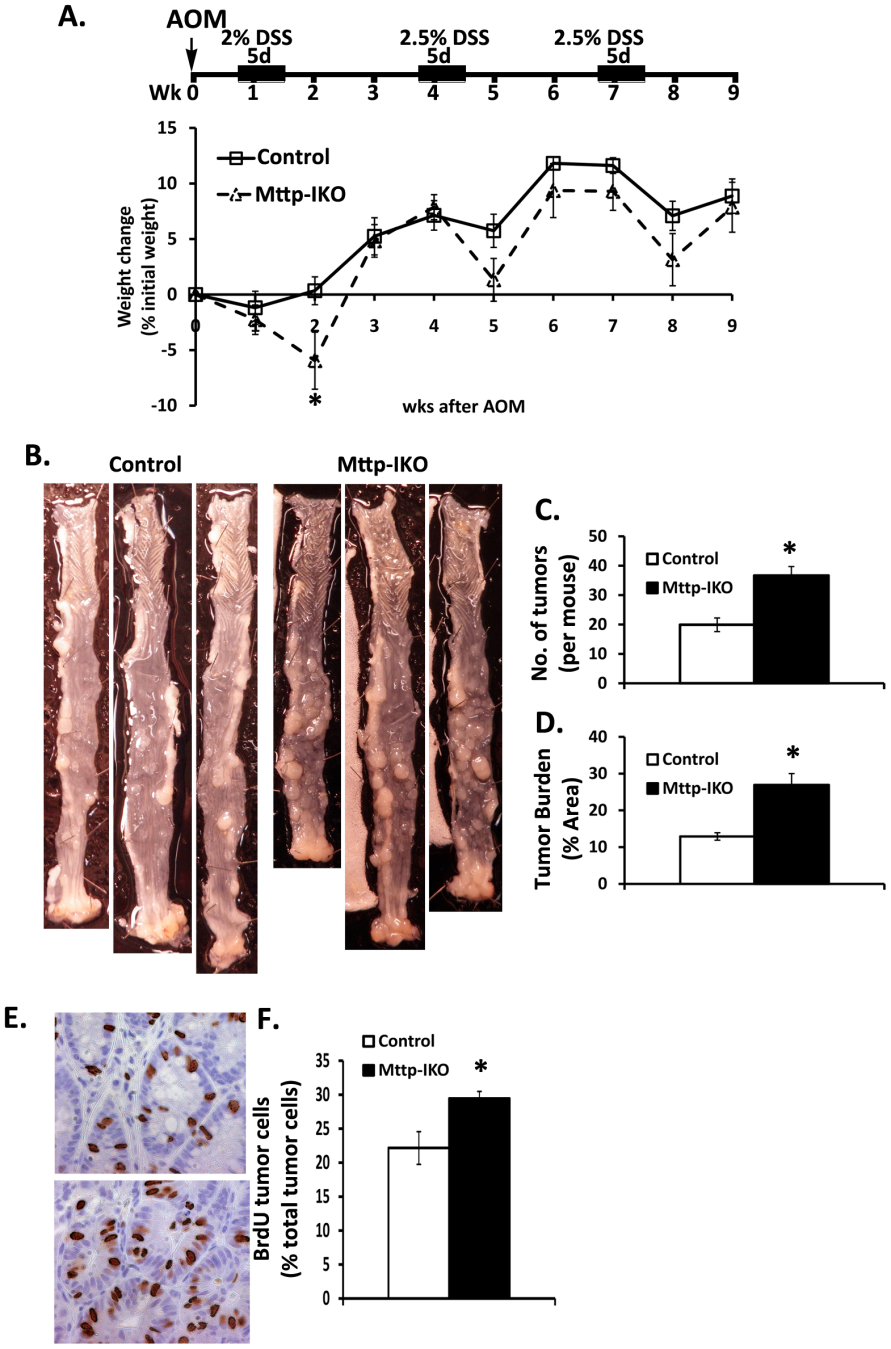
Increased formation of colitis-associated tumors in AOM/DSS treated*Mttp-IKO* mice. **A**. Upper panel: Schematic overview of the AOM/DSS protocol (detail in Methods). Mice were sacrificed 12 days after the last cycle of DSS. n = 13–15 mice each group. Lower panel: Percent weight change during AOM-DSS treatment. **B**. Representative colonic photos. **C**. Number of colorectal tumors per mouse induced by AOM-DSS treatment. **D**. Tumor burden in AOM-DSS treated control and *Mttp-IKO* mice. Data from panels D. are expressed as means ± SEM of tumor area normalized to the total colonic area, n = 9–10 mice each group. **E. and F**. Increased polyp proliferation by BrdU staining. (**E**) is a representative photo, upper panel is a control polyp, lower panel is from a *Mttp-IKO* polyp. (**F**) is the bar graph of mean ± SEM (n = 5–6 mice per group). * p<0.05.

A substantial body of data suggests that inflammation is a major driver of tumorigenesis and work suggests a causative link mediated via pathways involving inflammatory cytokine signaling [25], [26]. Given the propensity of *Mttp-IKO* mice to manifest a more severe phenotype in response to DSS mediated injury, we asked whether this increased colonic inflammation was associated with an altered colonic tumor burden following administration of azoxymethane followed by three cycles of DSS (Figure 6A). The findings reveal greater tumor multiplicity (Figure 6B) and overall tumor burden (Figure 6C, D) (verified by pathologic examination by a pathologist blinded to genotype) in *Mttp-IKO* mice in association with increased cellular proliferation as evidenced by BrdU incorporation (Figure 6E, F).

### Inflammatory signals coupled with altered fatty acid signaling drive growth mediator secretion in Mttp-IKO mice

**Figure 7:**
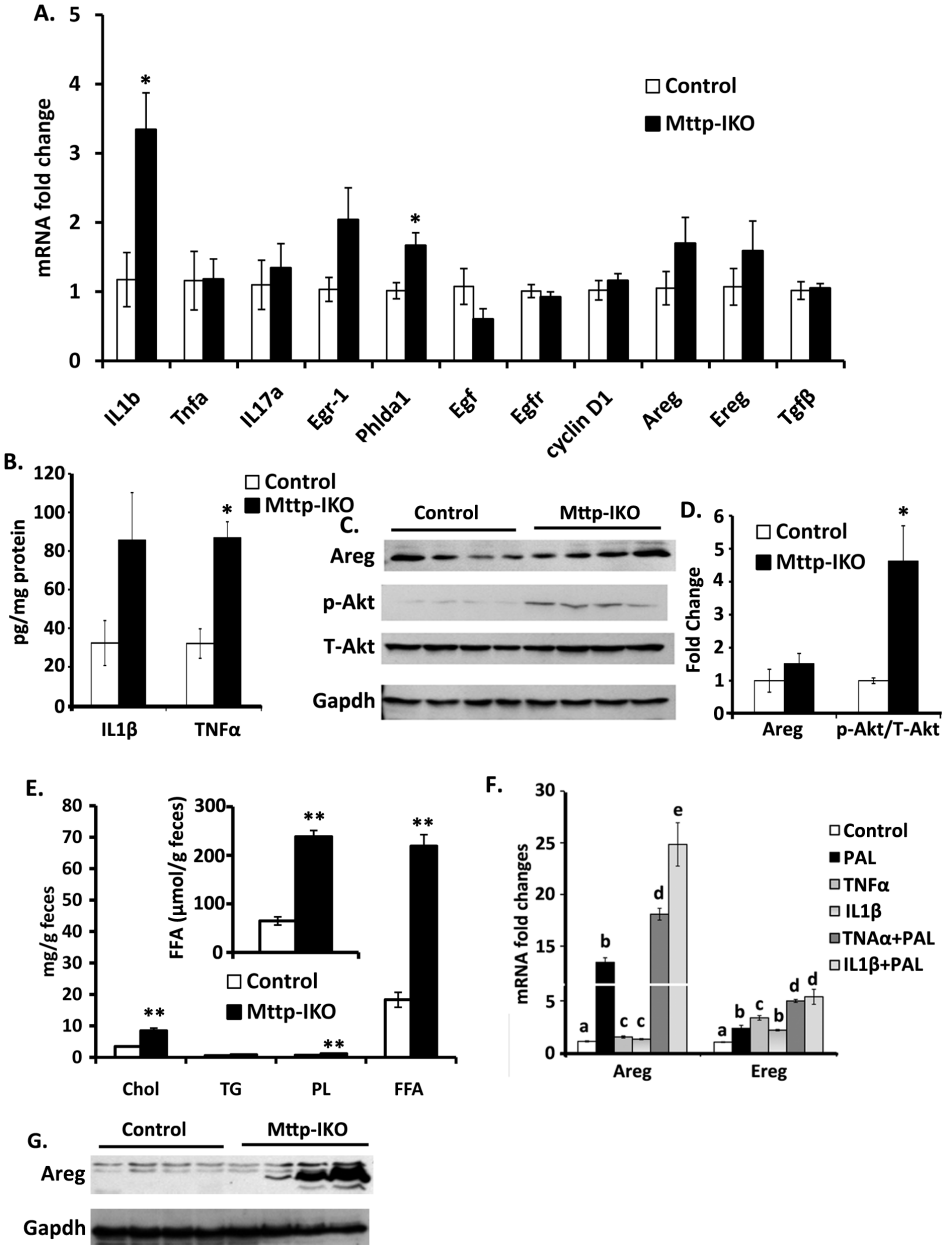
Altered production of growth mediators in*Mttp-IKO* mice. **A**. Altered mRNA expression of genes related to inflammation and growth. Gene expression in AOM/DSS-treated descending colon was measured by QPCR and expressed as fold change compared to control mice after normalization to Gapdh. Data are mean ± SEM (n = 4 each group). *p<0.05. **B**. Protein content of IL1β and TNFα in homogenates of descending colon from AOM/DSS-treated control and *Mttp-IKO* mice was measured by ElISA. Data are mean ± SEM (n = 4 each group). *p<0.05. **C**. Western blot analysis of amphiregulin (Areg), (1∶1000 dilution of primary antibody from Thermo Fisher Scientific, Cat#RB-258); Akt (1∶1000 of primary antibody from Cell Signaling, Cat#9272); p-Akt (1∶1000 of primary antibody from Cell Signaling, Cat# 9271) in homogenates of samples prepared (Methods) from tissues noted above in B. **D**. Western blot was quantitated by Kodak image station 440 and Carestream MI SE software. Bar graph reflects the mean ± SEM of relative expression compared to control after normalization to Gapdh. **E**. Fecal lipid species in chow-fed control and *Mttp-IK*O mice. Total fecal lipids were extracted and cholesterol (Chol), triglycerides (TG), phospholipids (PL), and free fatty acid (FFA) quantitated enzymatically. Lipid content is presented as µg/g feces and FFA content is also presented as µmol/g feces (inset). Data are mean ± SEM (n = 4–5 per group) **p<0.01. **F**. mRNA abundance of Areg and Ereg in primary intestinal myofibroblasts after the indicated stimulation. Myofibroblasts were stimulated with indicated factors for 12 hours. Gene (mRNA) expression was measured by QPCR and expressed as fold change compared to control cells after normalization to Gapdh. Experiments were performed twice with triplicates for each experiment. Data are presented as mean ± SEM. The difference between values by group is indicated by a different letter to indicate statistical significance (p<0.05 or greater). **G**. Western blot analysis of Areg protein expression in intestinal mucosa homogenates from control and *Mttp-IKO* mice on western diet for 2 weeks.

In order to understand in more depth the mechanisms underlying the proliferative phenotype observed in the colon of *Mttp-IKO* mice in response to AOM/DSS, we again examined the expression patterns of IL-1β and TNFα. We observed increased mRNA abundance of IL-1β in *Mttp-IKO* mice (Figure 7A) and a trend to increased IL-1β protein expression (Figure 7B). The expression of TNFα mRNA was comparable by genotype but we observed a significant increase in TNFα protein levels in colonic tissue from *Mttp-IKO* mice (Figure 7B). These findings again support the hypothesis that an altered inflammatory response likely contributes to the increased tumor burden observed in *Mttp-IKO* mice.

To explore the mechanisms at play in this response, we undertook an examination of possible mediators of this pro-proliferative phenotype. Transcript profiling revealed no significant changes in mRNA expression of several candidate mediators (Figure 7A), but there was a trend to increased amphiregulin protein expression and an increase in phospho-Akt abundance (Figure 7C, D). These findings suggested to us that the secretion of mediators from intestinal myofibroblasts might play a role in growth modulation via paracrine pathways. In support of this possibility there is work showing a role for stromal myofibroblast production of amphiregulin and epiregulin in promoting colon tumor growth in the setting of inflamed human colon [27]. Our approach was guided by earlier findings demonstrating that small intestinal epithelial cells from *Mttp-IKO* mice undergo lipotoxic injury following short term high fat feeding [22]. Based on those observations, we suspected that the sustained growth promoting effects of intestinal *Mttp* deletion were potentially mediated by myofibroblasts.

In order to explore this possibility in more detail, we turned to primary murine intestinal myofibroblasts in order to examine amphiregulin and epiregulin regulation. We first established that fecal lipid content was increased as a result of fat malabsorption in *Mttp-IKO* mice, as previously documented [11]. In particular, we demonstrated increased fecal free fatty acid (FFA) abundance as well as increased content of cholesterol and phospholipid in *Mttp-IKO* mice (Figure 7E). We determined that the fecal FFA concentration in control mice (∼60 µmol/g) was similar to that reported recently [28]. We focused our attention on the abundant saturated fatty acids, selecting palmitic acid as an initial target in which to examine the regulation of both amphiregulin and epiregulin [29]. Our findings demonstrate striking upregulation of amphiregulin and epiregulin mRNA in primary murine intestinal myofibroblasts by 200 µM palmitate (Figure 7E). Furthermore, the known induction of amphiregulin and epiregulin by TNFα and IL-1β [30] was further enhanced by the inclusion of palmitate (Figure 7E). These findings suggest that fatty acids play a role in modifying growth responses in conjunction with other known mediators. In line with this suggestion we demonstrated increased small intestinal expression of amphiregulin expression in *Mttp-IKO* mice following exposure to a high fat western diet (Figure 7F, G). Taken together, the findings strongly suggest that increased colonic fatty acid availability, produced by defective small intestinal chylomicron assembly and the consequent block in normal lipid absorption, exacerbates inflammatory and other adaptive pathways that increase colonic proliferation.

## Discussion

The findings in this report indicate that blocking chylomicron secretion from the small intestine is associated with increased severity of chemical (DSS) colitis and leads to increased tumor burden in a model of CAC. The mechanisms and pathways mediating these phenotypes include alterations in intestinal barrier function with increased permeability and increased serum levels of TNFα, coupled with activation of myofibroblast secretion of growth factors mediated at least in part through altered fatty acid signaling. Together the findings link aspects of intestinal lipid metabolism with intestinal inflammation and also to pathways that promote development of colorectal cancer. Some of these core observations merit more detailed discussion.

Previous studies have implicated lipid-derived factors secreted from the small intestine as key components in mediating distal organ failure in systemic sepsis [8], [9] although the precise lipid components and lipoprotein particles involved (chylomicrons, low or high density lipoprotein) are yet to be determined. Other work has demonstrated that bacterial LPS absorption by the small intestine involves chylomicron formation as evidenced by diminished serum LPS levels in animals treated with Pluronic L-81, which functions within enterocytes to block chylomicron formation [31]. Here we observed that serum LPS levels tended to increase following DSS treatment but we found no differences between control and *Mttp-IKO* mice. By contrast, we found elevated serum levels of TNFα in *Mttp-IKO* mice, both at baseline and following DSS treatment, suggesting that blocking chylomicron formation was associated with increased production or altered turnover of this proinflammatory cytokine in the setting of acute chemical colitis. Further work will be required to resolve the cellular origins of TNFα and to address the important questions of production and clearance rates of this cytokine in DSS mediated colonic injury. Nevertheless, our findings are consistent with other work that has implicated lipid-dependent alterations in intestinal delivery of dietary antigens that modulate systemic responses, including the release of inflammatory mediators from adipose tissue [32], [33].

Our recent findings demonstrated that *Mttp-IKO* mice were protected against pneumonia-associated sepsis induced intestinal injury and exhibited decreased mortality [12], which led us to speculate that blocking intestinal chylomicron secretion might actually be protective against other models associated with systemic inflammations. However, our findings demonstrated the opposite conclusion. Here we show that *Mttp-IKO* mice have a far worse outcome when challenged in a model of chemical (DSS) colitis. The apparent discordance in these outcomes lead us to suggest that blocking chylomicron assembly and secretion may protect animals from the secondary manifestations of intestinal injury only when the source of sepsis involves extraintestinal tissues (such as the lung). In the setting of pneumonia-associated sepsis, for example, *Mttp-IKO* mice exhibit a compensatory increase in hepatic HDL production [12]. By contrast, the current findings strongly imply that blocking chylomicron formation and decreasing dietary lipid absorption exacerbates the systemic effects of inflammatory stimuli associated with colonic injury.

Another consideration in attempting to understand the pathways by which blocking chylomicron secretion exaggerates the inflammation associated with colonic injury is that the genetic pathways involved in Mttp-dependent lipid delivery into the endoplasmic reticulum [34] overlap with Mttp-dependent pathways involved in lipid antigen processing. In this alternative setting, Mttp has been shown to play a key role in lipidating CD1 antigen presenting family members [35]. This is relevant because lipid antigen loading onto CD1d molecules is critical for presentation to natural killer T (NKT) cells [36]. In relation to the current findings, it is worth noting that human subjects with abetalipoproteinemia (ABL) (in whom the *MTTP* gene is defective) have been shown to exhibit defective CD1 function as a result of impaired lipid antigen loading [37]. It is also worth noting that while the incidence of colorectal cancer in ABL subjects is unknown, there is a case report of ileal adenocarcinoma in a patient with mild ABL [38]. Thus a confluence of findings suggest that Mttp may function in pathways beyond dietary lipid absorption and might conceivably play a role in the pathogenesis of intestinal inflammatory and protective processes mediated by CD1 dependent pathways [39]. In keeping with this suggestion, studies demonstrated that *Mttp* deletion was protective against experimental oxazalone-induced colitis, which is dependent on the presumptively haptenated antigen being presented via invariant NKT cells [40]. Those studies however did not address the specific role of intestinal epithelial *Mttp* deletion, but rather used an Mx-1 (interferon-inducible) Cre [40]. The question of whether intestine-specific *Mttp* deletion (as in the current study) exhibits the same protective phenotype as that observed using the interferon inducible system employed in those earlier studies will await clarification.

The current findings also raise the question of how dietary fat and intestinal lipid metabolism might influence inflammation and pathways relevant to the pathogenesis of colorectal cancer. It has been shown for example that mice chronically fed high fat diets exhibit increased mortality in some models of sepsis [41] while other studies demonstrated that high fat diets induce low grade endotoxemia in both experimental animals and in studies using intestinal cell culture [42]. Recent studies in otherwise healthy human subjects has further shown that feeding a western-style high fat diet produces features of systemic inflammation, including endotoxemia [43]. In keeping with these findings, other studies in obese subjects demonstrated that diet-induced weight loss led to reductions in serum cytokines associated with systemic inflammation (including TNFα, IL-1β), [44]. Taken together, the findings from in vitro studies together with studies using experimental animal models as well as human studies reinforce the concept that strategies including reducing dietary fat intake may attenuate the pro-inflammatory state associated with obesity and in turn reduce the risk of colorectal cancer. It bears emphasis that the current studies were undertaken in two genotypes of mice fed low fat rodent chow, suggesting that the increased fatty acid flux in *Mttp-IKO* mice resulted from the unabsorbed lipid rather than from increased dietary fat intake or from obesity (since *Mttp-IKO* mice are lean), or from increased colonic accumulation of neutral lipid. In this regard, we validated fecal FFA content in control, chow-fed mice (∼60 µmol/g, Figure 7E) was similar to that recently reported [28]. In addition, those authors demonstrated that palmitic acid represented the dominant saturated FA species found in feces [28]. Extrapolating from their findings, we estimate that palmitic acid is present in control, chow-fed mouse feces at a concentration of ∼20 millimolar [28]. These are important considerations in evaluating the effects of palmitic acid as used in the current studies. We selected a concentration (200 µM) previously shown in cell models to stimulate lipotoxic adaptive responses [29]. Our findings demonstrate a significant induction of epiregulin and amphiregulin expression in intestinal myofibroblasts, even at FFA concentrations log orders lower than found within the colonic lumen. Based upon these findings, it would be of interest to examine the effects if any of short-term exposure to high fat diets on colorectal cancer development or progression in *Mttp-IKO* mice.

Finally, our findings raise the possibility that there may be other adverse effects of *Mttp* deletion beyond lipid malabsorption. Pharmacologic compounds that block Mttp activity are currently in clinical trials, including some that are by design intestine-specific [45]–[49]. Our findings raise the concern that human subjects consuming these agents might be more susceptible to inflammatory conditions affecting the intestine and potentially also at greater risk of developing colorectal tumors.

## References

[1] Aleksandrova K, Nimptsch K, Pischon T (2013) Influence of Obesity and Related Metabolic Alterations on Colorectal Cancer Risk. Curr Nutr Rep 2: 1–9. 23396857 10.1007/s13668-012-0036-9 PMC3562548

[2] Yehuda-Shnaidman E, Schwartz B (2012) Mechanisms linking obesity, inflammation and altered metabolism to colon carcinogenesis. Obes Rev 13: 1083–1095. 22937964 10.1111/j.1467-789X.2012.01024.x

[3] Fung TT, Hu FB, Schulze M, Pollak M, Wu T, et al (2012) A dietary pattern that is associated with C-peptide and risk of colorectal cancer in women. Cancer Causes Control 23: 959–965. 22535146 10.1007/s10552-012-9969-y PMC3572718

[4] Clark JA, Coopersmith CM (2007) Intestinal crosstalk: a new paradigm for understanding the gut as the "motor" of critical illness. Shock 28: 384–393. 17577136 10.1097/shk.0b013e31805569df PMC2084394

[5] Hassoun HT, Kone BC, Mercer DW, Moody FG, Weisbrodt NW, et al (2001) Post-injury multiple organ failure: the role of the gut. Shock 15: 1–10. 10.1097/00024382-200115010-00001 11198350

[6] Deitch EA, Forsythe R, Anjaria D, Livingston DH, Lu Q, et al (2004) The role of lymph factors in lung injury, bone marrow suppression, and endothelial cell dysfunction in a primate model of trauma-hemorrhagic shock. Shock 22: 221–228. 15316391 10.1097/01.shk.0000133592.55400.83

[7] Deitch EA, Xu D, Kaise VL (2006) Role of the gut in the development of injury- and shock induced SIRS and MODS: the gut-lymph hypothesis, a review. Front Biosci 11: 520–528. 16146750 10.2741/1816

[8] Deitch EA (2010) Gut lymph and lymphatics: a source of factors leading to organ injury and dysfunction. Ann N Y Acad Sci 1207 Suppl 1E103–111. 20961300 10.1111/j.1749-6632.2010.05713.x

[9] Senthil M, Brown M, Xu DZ, Lu Q, Feketeova E, et al. (2006) Gut-lymph hypothesis of systemic inflammatory response syndrome/multiple-organ dysfunction syndrome: validating studies in a porcine model. J Trauma 60: :958-965;discussion 965–957. 10.1097/01.ta.0000215500.00018.47 16688055

[10] Neumann S, Coudreuse DY, van der Westhuyzen DR, Eckhardt ER, Korswagen HC, et al (2009) Mammalian Wnt3a is released on lipoprotein particles. Traffic 10: 334–343. 19207483 10.1111/j.1600-0854.2008.00872.x

[11] Xie Y, Newberry EP, Young SG, Robine S, Hamilton RL, et al (2006) Compensatory increase in hepatic lipogenesis in mice with conditional intestine-specific Mttp deficiency. J Biol Chem 281: 4075–4086. 16354657 10.1074/jbc.M510622200

[12] Dominguez JA, Xie Y, Dunne WM, Yoseph BP, Burd EM, et al (2012) Intestine-specific Mttp deletion decreases mortality and prevents sepsis-induced intestinal injury in a murine model of Pseudomonas aeruginosa pneumonia. PLoS One 7: e49159. 23145105 10.1371/journal.pone.0049159 PMC3493497

[13] Shaker A, Swietlicki EA, Wang L, Jiang S, Onal B, et al (2010) Epimorphin deletion protects mice from inflammation-induced colon carcinogenesis and alters stem cell niche myofibroblast secretion. J Clin Invest 120: 2081–2093. 20458144 10.1172/JCI40676 PMC2877942

[14] Greten FR, Eckmann L, Greten TF, Park JM, Li ZW, et al (2004) IKKbeta links inflammation and tumorigenesis in a mouse model of colitis-associated cancer. Cell 118: 285–296. 15294155 10.1016/j.cell.2004.07.013

[15] Rachmilewitz D, Karmeli F, Takabayashi K, Hayashi T, Leider-Trejo L, et al (2002) Immunostimulatory DNA ameliorates experimental and spontaneous murine colitis. Gastroenterology 122: 1428–1441. 11984528 10.1053/gast.2002.32994

[16] Kerr TA, Ciorba MA, Matsumoto H, Davis VR, Luo J, et al (2012) Dextran sodium sulfate inhibition of real-time polymerase chain reaction amplification: a poly-A purification solution. Inflamm Bowel Dis 18: 344–348. 21618356 10.1002/ibd.21763 PMC3600644

[17] Plateroti M, Rubin DC, Duluc I, Singh R, Foltzer-Jourdainne C, et al (1998) Subepithelial fibroblast cell lines from different levels of gut axis display regional characteristics. Am J Physiol 274: G945–954. 9612277 10.1152/ajpgi.1998.274.5.G945

[18] Swift LL, Jovanovska A, Kakkad B, Ong DE (2005) Microsomal triglyceride transfer protein expression in mouse intestine. Histochem Cell Biol 123: 475–482. 15891896 10.1007/s00418-005-0772-7

[19] Levy E, Stan S, Garofalo C, Delvin EE, Seidman EG, et al (2001) Immunolocalization, ontogeny, and regulation of microsomal triglyceride transfer protein in human fetal intestine. Am J Physiol Gastrointest Liver Physiol 280: G563–571. 11254482 10.1152/ajpgi.2001.280.4.G563

[20] Fasano A (2012) Intestinal permeability and its regulation by zonulin: diagnostic and therapeutic implications. Clin Gastroenterol Hepatol 10: 1096–1100. 22902773 10.1016/j.cgh.2012.08.012 PMC3458511

[21] Consortium UIG, Barrett JC, Lee JC, Lees CW, Prescott NJ, et al (2009) Genome-wide association study of ulcerative colitis identifies three new susceptibility loci, including the HNF4A region. Nat Genet 41: 1330–1334. 19915572 10.1038/ng.483 PMC2812019

[22] Xie Y, Luo J, Kennedy S, Davidson NO (2007) Conditional intestinal lipotoxicity in Apobec-1-/- Mttp-IKO mice: a survival advantage for mammalian intestinal apolipoprotein B mRNA editing. J Biol Chem 282: 33043–33051. 17855359 10.1074/jbc.M705386200

[23] Ozcan U, Yilmaz E, Ozcan L, Furuhashi M, Vaillancourt E, et al (2006) Chemical chaperones reduce ER stress and restore glucose homeostasis in a mouse model of type 2 diabetes. Science 313: 1137–1140. 16931765 10.1126/science.1128294 PMC4741373

[24] Yan F, Wang L, Shi Y, Cao H, Liu L, et al (2012) Berberine promotes recovery of colitis and inhibits inflammatory responses in colonic macrophages and epithelial cells in DSS-treated mice. Am J Physiol Gastrointest Liver Physiol 302: G504–514. 22173918 10.1152/ajpgi.00312.2011 PMC3311435

[25] Grivennikov SI, Karin M (2010) Inflammation and oncogenesis: a vicious connection. Curr Opin Genet Dev 20: 65–71. 20036794 10.1016/j.gde.2009.11.004 PMC2821983

[26] Grivennikov SI, Karin M (2011) Inflammatory cytokines in cancer: tumour necrosis factor and interleukin 6 take the stage. Ann Rheum Dis 70 Suppl 1i104–108. 21339211 10.1136/ard.2010.140145

[27] Nishimura T, Andoh A, Inatomi O, Shioya M, Yagi Y, et al (2008) Amphiregulin and epiregulin expression in neoplastic and inflammatory lesions in the colon. Oncol Rep 19: 105–110. 18097582

[28] van Diepen JA, Stienstra R, Vroegrijk IO, van den Berg SA, Salvatori D, et al (2013) Caspase-1 deficiency in mice reduces intestinal triglyceride absorption and hepatic triglyceride secretion. J Lipid Res 54: 448–456. 23160218 10.1194/jlr.M031963 PMC3588871

[29] Huang S, Rutkowsky JM, Snodgrass RG, Ono-Moore KD, Schneider DA, et al (2012) Saturated fatty acids activate TLR-mediated proinflammatory signaling pathways. J Lipid Res 53: 2002–2013. 22766885 10.1194/jlr.D029546 PMC3413240

[30] Inatomi O, Andoh A, Yagi Y, Bamba S, Tsujikawa T, et al (2006) Regulation of amphiregulin and epiregulin expression in human colonic subepithelial myofibroblasts. Int J Mol Med 18: 497–503. 16865236

[31] Ghoshal S, Witta J, Zhong J, de Villiers W, Eckhardt E (2009) Chylomicrons promote intestinal absorption of lipopolysaccharides. J Lipid Res 50: 90–97. 18815435 10.1194/jlr.M800156-JLR200

[32] Wang Y, Ghoshal S, Ward M, de Villiers W, Woodward J, et al (2009) Chylomicrons promote intestinal absorption and systemic dissemination of dietary antigen (ovalbumin) in mice. PLoS One 4: e8442. 20041190 10.1371/journal.pone.0008442 PMC2793525

[33] Wang Y, Li J, Tang L, Wang Y, Charnigo R, et al (2010) T-lymphocyte responses to intestinally absorbed antigens can contribute to adipose tissue inflammation and glucose intolerance during high fat feeding. PLoS One 5: e13951. 21085605 10.1371/journal.pone.0013951 PMC2978720

[34] Hussain MM, Rava P, Walsh M, Rana M, Iqbal J (2012) Multiple functions of microsomal triglyceride transfer protein. Nutr Metab (Lond) 9: 14. 22353470 10.1186/1743-7075-9-14 PMC3337244

[35] Kaser A, Hava DL, Dougan SK, Chen Z, Zeissig S, et al (2008) Microsomal triglyceride transfer protein regulates endogenous and exogenous antigen presentation by group 1 CD1 molecules. Eur J Immunol 38: 2351–2359. 18624350 10.1002/eji.200738102 PMC4132950

[36] McCarthy C, Shepherd D, Fleire S, Stronge VS, Koch M, et al (2007) The length of lipids bound to human CD1d molecules modulates the affinity of NKT cell TCR and the threshold of NKT cell activation. J Exp Med 204: 1131–1144. 17485514 10.1084/jem.20062342 PMC2118584

[37] Zeissig S, Dougan SK, Barral DC, Junker Y, Chen Z, et al (2010) Primary deficiency of microsomal triglyceride transfer protein in human abetalipoproteinemia is associated with loss of CD1 function. J Clin Invest 120: 2889–2899. 20592474 10.1172/JCI42703 PMC2912200

[38] Al-Shali K, Wang J, Rosen F, Hegele RA (2003) Ileal adenocarcinoma in a mild phenotype of abetalipoproteinemia. Clin Genet 63: 135–138. 12630961 10.1046/j.0009-9163.2002.00175.x

[39] Yu KO, Porcelli SA (2005) The diverse functions of CD1d-restricted NKT cells and their potential for immunotherapy. Immunol Lett 100: 42–55. 16083968 10.1016/j.imlet.2005.06.010

[40] Brozovic S, Nagaishi T, Yoshida M, Betz S, Salas A, et al (2004) CD1d function is regulated by microsomal triglyceride transfer protein. Nat Med 10: 535–539. 15107843 10.1038/nm1043

[41] Strandberg L, Verdrengh M, Enge M, Andersson N, Amu S, et al (2009) Mice chronically fed high-fat diet have increased mortality and disturbed immune response in sepsis. PLoS One 4: e7605. 19865485 10.1371/journal.pone.0007605 PMC2765728

[42] Laugerette F, Vors C, Geloen A, Chauvin MA, Soulage C, et al (2011) Emulsified lipids increase endotoxemia: possible role in early postprandial low-grade inflammation. J Nutr Biochem 22: 53–59. 20303729 10.1016/j.jnutbio.2009.11.011

[43] Pendyala S, Walker JM, Holt PR (2012) A high-fat diet is associated with endotoxemia that originates from the gut. Gastroenterology142: :1100–1101 e1102. 10.1053/j.gastro.2012.01.034 PMC3978718 22326433

[44] Pendyala S, Neff LM, Suarez-Farinas M, Holt PR (2011) Diet-induced weight loss reduces colorectal inflammation: implications for colorectal carcinogenesis. Am J Clin Nutr 93: 234–242. 21147860 10.3945/ajcn.110.002683 PMC3021422

[45] Kim E, Campbell S, Schueller O, Wong E, Cole B, et al (2011) A small-molecule inhibitor of enterocytic microsomal triglyceride transfer protein, SLx-4090: biochemical, pharmacodynamic, pharmacokinetic, and safety profile. J Pharmacol Exp Ther 337: 775–785. 21406547 10.1124/jpet.110.177527

[46] Mera Y, Odani N, Kawai T, Hata T, Suzuki M, et al (2011) Pharmacological characterization of diethyl-2-({3-dimethylcarbamoyl-4-[(4′-trifluoromethylbiphenyl-2-carbonyl) amino]phenyl}acetyloxymethyl)-2-phenylmalonate (JTT-130), an intestine-specific inhibitor of microsomal triglyceride transfer protein. J Pharmacol Exp Ther 336: 321–327. 20974698 10.1124/jpet.110.173807

[47] Hata T, Mera Y, Kawai T, Ishii Y, Kuroki Y, et al (2011) JTT-130, a novel intestine-specific inhibitor of microsomal triglyceride transfer protein, ameliorates impaired glucose and lipid metabolism in Zucker diabetic fatty rats. Diabetes Obes Metab 13: 629–638. 21362121 10.1111/j.1463-1326.2011.01387.x

[48] Hata T, Mera Y, Tadaki H, Kuroki Y, Kawai T, et al (2011) JTT-130, a novel intestine-specific inhibitor of microsomal triglyceride transfer protein, suppresses high fat diet-induced obesity and glucose intolerance in Sprague-Dawley rats. Diabetes Obes Metab 13: 446–454. 21255216 10.1111/j.1463-1326.2011.01368.x

[49] Mera Y, Odani N, Kawai T, Hata T, Suzuki M, et al (2011) Pharmacological characterization of diethyl-2-({3-dimethylcarbamoyl-4-[(4′-trifluoromethylbiphenyl-2-carbonyl)amino]p henyl}acetyloxymethyl)-2-phenylmalonate (JTT-130), an intestine-specific inhibitor of microsomal triglyceride transfer protein. J Pharmacol Exp Ther 336: 321–327. 20974698 10.1124/jpet.110.173807

